# Effect of *Helicobacter pylori* Eradication on Serum Level of Valproic Acid in Children with Idiopathic Generalized Epilepsy

**DOI:** 10.3390/children11101259

**Published:** 2024-10-18

**Authors:** Abobakr Abdelgalil, Doaa Ismail, Ayman Eskander, Marian Girgis, Ahmed Farouk, Fajr Saeedi, Mohamed Shazly, Amera Hasnoon

**Affiliations:** 1 Department of Pediatrics, Faculty of Medicine, Cairo University, Cairo 12613, Egypt; dr.doaaatef@hotmail.com (D.I.); aymanemil@cu.edu.eg (A.E.); marian.girgis@kasralainy.edu.eg (M.G.); amera.hasnoon@kasralainy.edu.eg (A.H.); 2 Department of Clinical Pathology, Military Medical Academy, Cairo 12613, Egypt; rouka_ahmed@yahoo.com; 3 Department of Pediatrics, Faculty of Medicine in Rabigh, King Abdulaziz University, Jeddah 21589, Saudi Arabia; fasaeedi@kau.edu.sa; 4 Department of Pediatrics, King Abdulaziz University Hospital, Jeddah 21589, Saudi Arabia; mshthly@kau.edu.sa; 5 Department of Pediatrics, Mallwi Hospital, Minia 61631, Egypt

**Keywords:** *H. pylori*, eradication, epilepsy, valproic acid

## Abstract

Background/Objectives: The purpose of this study was to determine the influence of *H. pylori* eradication on the serum level of the orally administered valproic acid (VPA) in children with idiopathic generalized epilepsy; Methods: This prospective cohort observational study included 100 children with idiopathic generalized epilepsy, recruited from a neurology clinic from May 2021 to December 2021. The patients were divided into two groups, each containing 50 children. The first group had a positive *H. pylori* stool antigen and *H. pylori*-related symptoms, while the second group had a negative antigen. *H. pylori* Eradication therapy was given to the positive *H. pylori* group. The serum level of VPA was obtained at baseline and 4 weeks after eradication therapy. Results: Despite there being no significant difference between the *H. pylori*-positive and *H. pylori*-negative groups regarding the baseline VPA serum level (79.9 ± 13.9 and 77.9 ± 13.1 mcg/mL), respectively, the serum VPA level had significantly increased after *H. pylori* eradication therapy (99.4 ± 11 mcg/mL) (*p* value = 0.000), as opposed to the *H. pylori*-negative group (85.3 ± 10.9 mcg/mL) (*p* value = 0.142). Furthermore, there was a statistically significant association with a negative correlation between the VPA serum level after eradication and the number of epileptic attacks per month (*p* value = 0.033, R value = −0.301) and the dose of VPA (*p* value = 0.046, R value = −0.284). Conclusions: The eradication of *H. pylori* resulted in a highly significant improvement in the serum level of the orally given VPA in children with idiopathic generalized epilepsy, as well as an indirect decrease in the frequency of epileptic events per month, allowing for dose reduction. Eradication therapy may have anticonvulsant properties and might indirectly aid in the management of epileptic activity. *H. pylori* screening for children with idiopathic generalized epilepsy can optimize serum VPA levels, potentially leading to better seizure control. To our knowledge, this is the first study in the literature to describe the effect of *H. pylori* eradication on the serum level of the orally administered VPA in children with idiopathic generalized epilepsy.

## 1. Introduction

*Helicobacter pylori* (*H. pylori*) infection is the most common cause of chronic gastritis and variably leads to serious global public health concerns. It is usually acquired during childhood and can last a lifetime if left untreated. It affects about half of the world’s population, though prevalence varies depending on geography and sanitary practices, being higher in developing, low-income populations like African countries [1].

The majority of infected children can remain asymptomatic for a very long time. Consequently, chronic *H. pylori* colonization can cause diverse gastrointestinal (GI) tract pathologies as chronic gastritis, gastric or duodenal ulcers, and gastric cancers, especially gastric mucosa-associated lymphoid tissue lymphoma [2]. Furthermore, extra-gastrointestinal manifestations were reported, including hematological, dermatological, ophthalmologic, metabolic, neurological, autoimmune, and allergic disorders. Several pathogenic mechanisms have been proposed, such as the occurrence of molecular mimicry and the production of inflammatory status [3].

*H. pylori* infection has been linked to several neurological problems; however, most reports have yielded conflicting results [4]. An association between *H. pylori* and epilepsy has been reported. *H. pylori* could indirectly affect the brain function due to the release of cytokines such as IL-1, IL-6, and TNF-α, which disrupt the blood–brain barrier (BBB) by altering its tight junction proteins. This disruption allows for the passage of immune cells and potentially harmful substances into the brain, contributing to neuronal inflammation and neuronal damage. In addition, *H. pylori* infection induces the release of a variety of neurotransmitters, including dopamine, serotonin, adrenaline, and acetylcholine. Likewise, *H. pylori* infection could result in axonal/neuronal damage, the generation of free radicals, and alterations in the expression of neuropeptides such vasoactive intestinal peptide (VIP) expression. The increased permeability of the BBB and subsequent neuronal inflammation can exacerbate seizure activity. Inflammatory cytokines can directly affect neuronal excitability and synaptic function, leading to an increased risk of seizures. Additionally, the infiltration of immune cells into the brain parenchyma can further amplify the inflammatory response, creating a feedback loop that promotes epileptogenesis. *H. pylori* infection can also change the gut microbiome, which has a pertinent role in modulating the microbiome–gut–brain axis. *H. pylori*-induced dysbiosis can lead to the production of neuroactive metabolites and can further influence CNS inflammation and seizure susceptibility [5,6,7]. There might be another possible association between *H. pylori* infection and the poor control of neurological disorders via the impaired absorption of drugs used to treat these disorders [8].

*H. pylori* infection affects stomach physiological functions, especially affecting gastric acid secretion and GI motility. Additionally, *H. pylori* infection indirectly influences gastrointestinal functions by influencing the brain–gut axis. These changes can also affect drug absorption, necessitating the careful evaluation of oral compounds, particularly those with pH-dependent solubility [9]. *H. pylori* infection and altered drug absorption were investigated in individuals receiving several oral medications like iron, thyroxine, and L-dopa replacement therapy and revealed an existing inverse association between the degree of gastritis and indices of drug absorption, as well as an improvement in drug absorption following *H. pylori* eradication [10].

Until recently, little attention has been paid to the link between the absorption and efficacy of the commonly used anti-epileptic drugs in epileptic children harboring *H. pylori* infection and the effect of eradication therapy on the serum level of these commonly used drugs. Previous research demonstrated that some antiepileptic medications like phenytoin and carbamazepine could have altered absorption when administered with continuous enteral nutrition, which can be extrapolated to other GI conditions like infection or inflammation [11,12,13]. Also, special attention to drugs that require an acidic environment for absorption, such as carbamazepine and phenytoin, is needed, particularly in the context of factors that affect GI function, such as infection, inflammation, bariatric surgery, as well as medications that affect gastric PH like proton pump inhibitors (PPI). Of these anti-epileptic drugs, there is valproic acid (VPA), which is a broad-spectrum antiepileptic medication that is effectively used to treat a wide range of seizures and epileptic syndromes. VPA is commonly administered orally; therefore, absorption from the gastrointestinal tract is critical; also, it has a limited therapeutic range. Given that VPA is a weak acid and its absorption is highly dependent on the pH and integrity of the GI tract, it is plausible that *H. pylori* infection, which alters these factors, would impact VPA absorption. Our study fills a critical gap by providing empirical evidence on this specific interaction. Understanding the effect of *H. pylori* eradication on VPA levels may have significant clinical implications, including optimizing drug dosing and improving seizure control in patients with epilepsy. This study lays the groundwork for further research on the interplay between GI health and antiepileptic drug efficacy. Thus, it is very important to assess the sufficiency and predictability of its absorption for effective treatment [14].

The aim of this study was to determine the effect of *H. pylori* eradication on the serum level of the orally administrated VPA in children with idiopathic generalized epilepsy. To our knowledge, this is the first study in the literature describing the effect of *H. pylori* eradication on the serum level of the orally administered VPA in children with idiopathic generalized epilepsy.

## 2. Materials and Methods

This prospective cohort observational study was conducted at Al-Galaa Military Medical Hospital, Cairo, Egypt. Our research followed the ethical standards mentioned in the Declaration of Helsinki and was approved by the local ethical committee (MD-181-2021). Consent for study inclusion was obtained. This study included 100 children with idiopathic generalized epilepsy recruited from a pediatric neurology clinic and referred (as a combined care) to a pediatric GI clinic during the period of May 2021 to December 2021.

The inclusion criteria were children under the age of 14 years old who were diagnosed with idiopathic generalized epilepsy, according to ILAE (The International League Against Epilepsy) [15], based on their clinical presentation (generalized tonic–clonic seizures, absences, or myoclonic jerks) and their EEG showing spike-wave complexes and/or polyspikes, and were receiving valproic acid (immediate release tablet or syrup) therapy as a single anti-epileptic drug for at least 1 month without any change in the anti-epileptic regimen during the study period. The patients were divided into two groups, each including 50 children. The first group included those with a positive *H. pylori* stool antigen test (SAT) and who were also symptomatic for *H. pylori*-related GI ulceration symptoms or extra GI symptoms, and the other group included those with a negative *H. pylori* SAT.

Children with epilepsy due to secondary causes or those who had received a proton pump inhibitor (PPI) within two weeks or antibiotics within four weeks before *H. pylori* stool antigen testing were excluded. Also, asymptomatic patients with positive *H. pylori* SAT were excluded. Furthermore, patients who had a positive *H. pylori* infection but failed to eradicate the infection (a persistently positive *H. pylori* SAT) were also excluded.

Demographic data, history (including type of epilepsy, age at diagnosis of epilepsy, dose of VPA, and epileptic activity as the number of epileptic attacks per month, in addition to general and GI history), and examinations (including general and neurological examinations) were obtained during the clinic visits. Changes in weight, appetite, and activity were assessed by measuring the patient’s weight before starting the *H. pylori* eradication therapy and then after eradication during follow-up visits under consistent conditions, while appetite and activity were assessed by using patient self-reports. The patients were asked to complete a standardized questionnaire at baseline (before eradication therapy) and at follow-up (after eradication therapy). The questionnaire included specific questions about their appetite and activity levels, with response options indicating whether these parameters had increased, decreased, or remained unchanged. Also, laboratory investigations including CBC, iron, ferritin, and TIBC were obtained. The detection of an *H. pylori* SAT by the enzyme immunoassay monoclonal antibody-based ELISA test as well as the serum level of VPA had been obtained at baseline, and again, both the *H. pylori* SAT and serum level of VPA were repeated 4 weeks after the completion of the therapy to ensure eradication and to detect changes in the serum level of VPA.

Regarding the *H. pylori* SAT assay, the ratio of 1:3 serially diluted calibrators was prepared using *H. pylori* Ag Calibrator Level 6 and Assay Buffer as the dilution buffer. Patient samples also need to be diluted 1:24 with Assay Buffer, with the centrifugation of the diluted fecal sample at 3000 rpm for 5–10 min. The *H. pylori* antigen in the stool assay utilized the microplate-based enzyme immunoassay technique by coating a highly purified monoclonal *H. pylori* antibody onto the wall of microtiter wells. The *H. pylori* antigen, if present in fecal specimens, could be bound to the antibody-coated plate after an incubation period. The unbound material was washed away, and another Horseradish Peroxidase (HRP)-conjugated monoclonal antibody which specifically recognizes the protein of *H. pylori* was added for further immunoreactions. After an incubation period, the immune complex of “*H. pylori* Antibody—*H. pylori* Antigen—HRP-conjugated Anti-*H. pylori* Tracer Antibody” was formed if the *H. pylori* antigen was present in the test sample. The unbound tracer antibody and other proteins in the buffer matrix were removed in the subsequent washing step. The HRP conjugated tracer antibody bound to the well was then incubated with a substrate solution, then measured in a spectrophotometric microplate reader by reading the absorbance at 450/620 nm,with calculation of the average absorbance for each pair of duplicate test results, followed by subtraction of thisaverage from the average absorbance of all other readings to obtain the corrected absorbance. The enzymatic activity of the tracer antibody bound to the *H. pylori* proteins captured on the wall of each microtiter well was directly proportional to the amount of the *H. pylori* antigen level in each test specimen (Epitope Diagnostics, Inc., San Diego, CA, USA).

Regarding the serum VPA assay, the serum levels of sodium valproate were determined by the HPLC-UV method. An equivalent amount of acetonitrile with 2.5 μg diazepam—as an internal standard—was added to a 1 mL patient blood sample and then centrifuged at 4000 rpm for 10 min. Then, 20 μL of the supernatant was injected using Hamilton injection into HPLC. Chromatograms of the individual subjects were recorded. The therapeutic range for VPA is 50–100 mcg/mL.

*H. pylori* eradication therapy was given to the positive *H. pylori* group in a single arm, open-label design protocol by a single investigator. The regimen was a combination therapy of a high dose of PPI (omeprazole) (2 mg/kg/day), amoxicillin (50 mg/kg/day), and metronidazole (30 mg/kg/day) in the form of tablets or suspension in young children who cannot swallow the tablet form for 2 weeks according to the ESPGHAN/NASPGHAN 2016 updated guidelines [16]. The change in the VPA dose after eradication therapy and the number of epileptic attacks were followed in the clinic to determine the effect of the eradication of *H. pylori* in the epilepsy course and the anti-epileptic VPA level.

The sample size of 100 children was chosen based on the given prevalence of *H. pylori* infection and idiopathic generalized epilepsy in the pediatric population. We aimed to recruit a sample size that was feasible within the study period (May 2021 to December 2021) while ensuring a representative sample. There was no previous study examining the impact of *H. pylori* eradication on VPA absorption in children with idiopathic generalized epilepsy. The sample size was calculated by using the PASS11 program for sample size calculation and assuming the proportion of increase in the level of the orally administered drug as VPA after *H. pylori* eradication = 50%. The sample size of 50 patients in each group can detect differences with a power of 80% and α-error of 0.05.

The statistical package for the social sciences (SPSS) version 26 was used to code and enter the data (IBM, Armonk, NY, USA). Quantitative data were represented as the mean, standard deviation, median, minimum, and maximum. The outcome of each measurement was correlated using Pearson’s correlation. Serum VPA levels were compared by two independent sample t tests. Statistical significance was set at a *p*-value of <0.05.

## 3. Results

This study included 100 patients known to have idiopathic generalized epilepsy, with a mean (SD) age at the time of inclusion in this study of 5 ± 1.8 years; Sixty-three (63%) patients were females. The mean age at first diagnosis of epilepsy was 3.5± 1.2 years; seventy-one (71%) patients exhibited the generalized tonic clonic type of seizures. Demographic and baseline characteristics are shown in Table 1.

The patients were divided into two groups; each included 50 children: the *H. pylori*-positive group and *H. pylori* negative infection. Comparing both groups, there was no statistically significant difference regarding age, gender, age of onset, and frequency of the epileptic attacks. Forty-one patients (82%) in the *H. pylori* positive group and thirty patients (60%) in the negative *H. pylori* group predominately exhibited the generalized tonic clonic type of seizures.

Also, there was a statistically significant association between the *H. pylori*-positive group and GIT manifestations. Half (50%) of the *H. pylori*-positive group had abdominal pain, mainly epigastric and related to meals, as opposed to only 24% of the *H. pylori*-negative group (*p* value = 0.007). Moreover, nausea and vomiting were significantly reported in 22% and 36% of the *H. pylori*-positive group compared to only 4% and 2% of the *H. pylori*-negative group (*p* value = 0.007 and 0.000, respectively).

Furthermore, more than half of *H. pylori*-positive patients reveled significant extra GI manifestations like hair changes in the form of dry hair and loose/fall hair (*p* value = 0.004 and 0.028, respectively). Also, *H. pylori*-positive patients revealed a significant iron deficiency anemia (*p* value = 0.000), but no significant difference was noted regarding other hematological parameters. A comparison between both groups is summarized in Table 2.

Despite there being no significant difference between the *H. pylori*-positive and *H. pylori*-negative groups regarding the baseline VPA serum level (79.9 ± 13.9 and 77.9 ± 13.1 mcg/mL, respectively), the serum VPA level significantly increased after *H. pylori* eradication therapy (99.4 ± 11 mcg/mL) (*p* value = 0.000), as opposed to the *H. pylori*-negative group (85.3 ± 10.9 mcg/mL) (*p* value = 0.142), indicating significant improvement in the serum VPA level after receiving the eradication therapy for *H. pylori* infection. The VPA serum level before and after eradication therapy is illustrated in Table 3.

Additionally, Following *H. pylori* eradication therapy, a significant improvement in the parameters of weight, appetite, and activity had been detected in almost half of the *H. pylori*-positive patients after the eradication of the *H. pylori* infection (*p* value= 0.016, 0.005, and 0.025 respectively) (Table 4).

Correlations between the VPA serum level % change and different parameters (epidemiological ones, weight, appetite, and activity changes) are provided in the Supplementary Material (Tables S1–S4).

**Figure 1:**
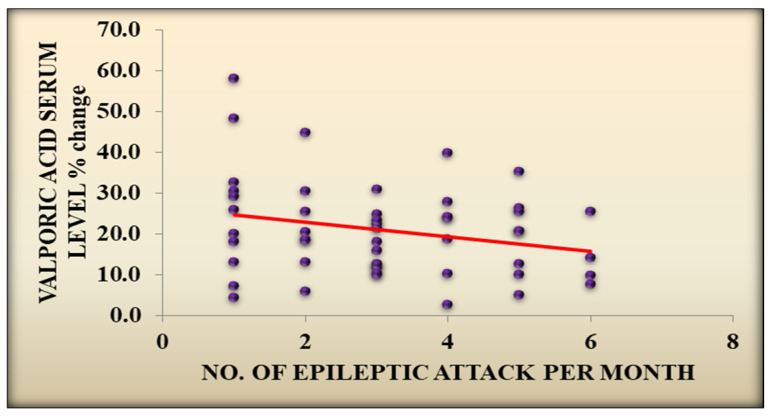
VPA serum level after eradication and change in number of epileptic attacks/month.

**Figure 2:**
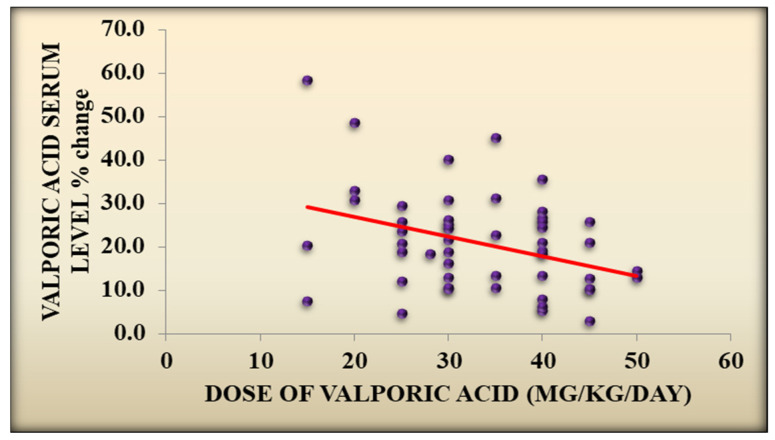
VPA serum level after eradication and change in dose of VPA.

Likewise, there was a statistically significant association between *H. pylori* eradication and a reduction in the number of epileptic attacks per month, with a median IQR of 3 (2–4) before eradication and a median IQR of 2 (1–2) after eradication (*p* value < 0.001) and with a statistically significant association with a negative correlation between the VPA serum level change after eradication and the number of epileptic attacks per month (*p* value = 0.033, R value = −0.301), as well as the dose of VPA (*p* value = 0.046, R value = −0.284) in the *H. pylori*-positive treated group, Figure 1 and Figure 2 and Supplementary Material Table S5.

## 4. Discussion

*H. pylori* infection, the most common cause of chronic gastritis, can lead to serious gastroduodenal diseases like peptic ulcers and gastric cancer. Also, variable extra-gastrointestinal hazards are well attributed to *H. pylori* infection. Typically acquired in childhood, *H. pylori* affects 50% of the global population and causes multiple phenotypes [1]. *H. pylori* infection has been linked to multiple extra-GI disorders; of these, several neurological problems like epilepsy have been reported, either via cytokine-mediated neuronal inflammation [5] or possibly owing to the impaired absorption of drugs used to treat these neurological disorders [8].

Until recently, little attention has been devoted to the relationship between the absorption of anti-epileptic drugs in epileptic children infected with *H. pylori* and how the eradication of this infection can affect absorption and serum drug levels. Among these anti-epileptic medications, there is VPA, which is a broad spectrum antiepileptic drug, usually given orally, so the absorption from the gastrointestinal tract is crucial [14].

The aim of this study was to determine the effect of *H. pylori* eradication on the serum level of the orally administered VPA in children with idiopathic generalized epilepsy.

In this cohort, the 100 patients with idiopathic generalized epilepsy were divided into two groups: those with and without *H. pylori* infection. Comparing both groups, although there was no significant difference in age, gender, and onset or frequency of epileptic attacks between both groups, a significant association was found between *H. pylori* positivity and GIT manifestations, especially abdominal pain, nausea, and vomiting, which were exhibited in 50%, 22%, and 36% (*p* values = 0.007, 0.007 and 0.000, respectively), in agreement with the previous research [17]. *H. pylori* infection is known to disrupt some physiological activities of the stomach, including gastric acid secretion and GI motility. About one-third of *H. pylori* patients have antral hypo-motility and delayed stomach emptying [9].

Besides GI manifestations, more than half of *H. pylori*-positive patients revealed significant extra GI manifestations like hair changes in the form of dry hair and loose/fall hair (*p* values = 0.004 and 0.028, respectively). Also, the *H. pylori*-positive group revealed hematological disorders and obviously significant iron deficiency anemia (*p* value = 0.000). Several reports suggest a link between *H. pylori* and extra-GI manifestations, including hematological, dermatological, ophthalmologic, metabolic, neurological, autoimmune, and allergic disorders. Several pathogenic mechanisms have been proposed, such as the occurrence of molecular mimicry and the production of an inflammatory status. Previous reports stating a potential association between *H. pylori* and hair changes like the loss of hair were published, with patients experiencing remission after eradication treatment. Furthermore, several meta-analyses revealed a strong relationship between *H. pylori* infection and iron deficiency anemia, with variable underlying pathophysiologic mechanisms like bleeding from ulcers, the impairment of iron absorption, the induction of pro-inflammatory cytokines, and gene polymorphisms [3,18,19].

Although there was no significant difference in baseline VPA serum levels between the *H. pylori*-positive and -negative groups (79.9 ± 13.9 and 77.9 ± 13.1 mcg/mL, respectively), the serum VPA levels significantly increased after *H. pylori* eradication therapy (99.4 ± 11 mcg/mL) (*p* value = 0.000), compared to the *H. pylori*-negative group (85.3 ± 10.9 mcg/mL) (*p* value = 0.142), indicating a significant improvement in serum drug levels after the eradication. In accordance with the present findings, previous studies have demonstrated that *H. pylori* infection can change stomach function, including gastric acid secretion and GI motility. Inflammation in the antral area leads to increased gastrin production. Severe inflammation in the corpus can significantly impair gastrin secretion. Approximately 30% of patients with *H. pylori* also have antral hypo-motility and delayed gastric emptying, which may be attributed to *H. pylori*-induced myoelectric activity variations. Delayed gastric emptying may cause alterations in the typical pathway of drug absorption by encouraging bacterial overgrowth, which can compete with absorption mechanisms and increase the contact time between a substance and gastric enzymes. Moreover, *H. pylori* infection causes the disruption of the stomach barrier, leading to the inflammation of gastric mucosa with the impairment of gastric physiology and the disruption of mucosal integrity. During the initial phase of infection, inflammatory products may directly decrease the production of parietal cells. Gastric acid secretion is inhibited by interleukin 1β, a pro-inflammatory cytokine and a major mediator in *H. pylori*-associated disease. After the elimination of *H. pylori*, gastric hypoacidity may be partially or completely recovered without sacrificing the majority of parietal cells. Long-term *H. pylori* injury may result in a significant loss of parietal cells, the atrophy of oxyntic glands present in the corporal mucosa, and the hypoacidity of the gastric juice. Furthermore, *H. pylori* eradication may change the gut microbiome composition, which may affect drug metabolism and absorption. *H. pylori* infection may indirectly alter GI function by affecting the brain–gut axis. This can affect other organs and disrupt permeability, motility, and secretion through the GI tract, not only the stomach. These alterations can significantly affect the absorption of oral medications, leading to important clinical implications, so oral medications and formulations with pH-dependent solubility should be carefully evaluated when supplied to *H. pylori*-positive individuals [9,20,21], as seen with thyroxine, whose absorption was impaired in *H. pylori* infection due to the related atrophic gastritis that may change the ionization status and the structural properties of the thyroxine molecule and thus change the efficiency of its intestinal absorption [22]. Another example is L-dopa; *H. pylori* can affect L-dopa bioavailability through the disruption of the mucosal integrity of the duodenum, the site of L-dopa absorption, and reactive oxygen species production. Also, gastric acid hypersecretion may reduce the solubility and absorption. The eradication of infection improves L-dopa bioavailability and clinical outcomes [23]. Additionally, the commonly used medication in the pediatric population is iron, which is also affected by *H. pylori* infection via several mechanisms like reduced acid secretion and the inhibition of ascorbic acid secretion, which interferes with the transformation of iron to the absorbable ferrous form. Furthermore, *H. pylori*-induced gastric inflammation can lead to increased levels of derivatives that bind to iron and increase iron loss in stool [24].

Similar pathophysiologic mechanisms like GI inflammation and a change in gastric acidity induced by *H. pylori* could affect VPA serum levels, with a significant improvement in serum levels after eradication therapy. VPA is primarily absorbed in the small intestine, where the presence of *H. pylori* can significantly alter the local environment. *H. pylori* infection can lead to changes in gastric pH, mucosal integrity, and motility, all of which can impact the absorption of drugs like VPA that rely on a stable GI environment. Also, VPA is a weak acid, and its absorption is highly dependent on the pH of the GI tract. *H. pylori* infection often alters gastric pH, which can affect the solubility and, consequently, the absorption of VPA. Moreover, *H. pylori* infection can cause chronic gastritis and damage to the gastric mucosa, leading to impaired drug absorption. The eradication of *H. pylori* can restore mucosal integrity, thereby improving the absorption of VPA. Additionally, *H. pylori* infection can affect GI motility, leading to delayed gastric emptying and altered drug transit times. These changes can impact the absorption kinetics of VPA more significantly than other antiepileptic drugs that may have different absorption profiles. Additionally, studies have shown that GI conditions, including infections and inflammation, can significantly impact the absorption and efficacy of orally administered medications. VPA, due to its specific absorption characteristics, is particularly sensitive to these changes, making it an ideal candidate for studying the effects of *H. pylori* eradication on its absorption. In patients with epilepsy, GI physiopathology might contribute to the epileptic disorder. Drug interventions that restore normal gut function may have anticonvulsant benefits. Furthermore, it should be noted that an alteration of GI function can reduce VPA antiepileptic pharmacological effects, and a thorough clinical evaluation should always be conducted [9,10,25,26,27].

Additionally, following *H. pylori* eradication therapy, a significant improvement in the parameters of weight, appetite, and activity had been detected (*p* values = 0.016, 0.005, and 0.025, respectively). *H. pylori* infection affects nutrients and drug absorption, hormone synthesis, and growth regulation. It impairs iron and vitamin B12 absorption. Also, infected individuals have lower ghrelin levels and higher leptin concentrations, which negatively affect growth and appetite [28], but some authors found no significant difference in circulating ghrelin levels before and after *H. pylori* eradication [29].

Furthermore, there was a statistically significant association with a negative correlation between the VPA serum level after eradication and the number of epileptic attacks per month (*p* value = 0.033, r value = −0.301) and the dose of VPA (*p* value = 0.046, r value = −0.284). Eradication therapy that restores normal gut function may have anticonvulsant benefits and can indirectly help in controlling epileptic activity [25].

The main limitations of our study included the lack of gastroduodenal histopathology- and endoscopy-based testing for *H. pylori* (gastric culture/PCR; histology) and also the lack of an antibiotic susceptibility test; so, the reliance on stool antigen testing could have the potential for false positives or negatives, which can impact the accuracy of the diagnosis regarding their *H. pylori* status. This misclassification could affect the study’s findings on the relationship between *H. pylori* eradication and VPA serum levels. However, we tried to deal with this limitation by using the most accurate method of the *H. pylori* stool antigen detection, the enzyme immunoassay monoclonal antibody-based ELISA, which is highly sensitive and specific and also non-invasive, particularly in the symptomatic study patients; also, in an attempt to increase the accuracy of the test, patients who had received a PPI within two weeks or antibiotics within four weeks before *H. pylori* SAT were excluded. Moreover, asymptomatic patients with a positive *H. pylori* stool antigen were excluded. Furthermore, patients with a positive *H. pylori* infection but who failed to eradicate the infection (a persistently positive *H. pylori* SAT) were excluded. Nevertheless, histologic diagnosis remains the gold standard for diagnosing *H. pylori* infection in children. Future studies should consider using histological confirmation or combining multiple diagnostic methods to improve accuracy. Another limitation is the short follow-up period of 4 weeks post-eradication; a longer follow-up period would also allow for the observation of any sustained improvements in seizure control and potential adjustments in VPA dosage over time. This information is crucial for optimizing treatment strategies and ensuring the best possible outcomes for children with idiopathic generalized epilepsy. By acknowledging these limitations and their potential impact on the study’s findings, we aim to provide a more comprehensive understanding of the results and suggest directions for future research.

## 5. Conclusions

In conclusion, the eradication of *H. pylori* resulted in a highly significant improvement in the serum level of the orally given VPA in children with idiopathic generalized epilepsy, with an indirect decrease in the frequency of epileptic events per month, allowing for antiepileptic dose reduction. Eradication therapy may have anticonvulsant properties and might indirectly aid in the management of epileptic activity. *H. pylori* screening for children with idiopathic generalized epilepsy can optimize serum VPA levels, potentially leading to better seizure control. This can be accomplished using non-invasive methods such as stool antigen tests. If indicated, eradication therapy should be considered as part of epilepsy management, with regular follow-up appointments to assess patient response and make necessary adjustments. Collaboration between pediatric neurologists and gastroenterologists is crucial for *H. pylori* screening and eradication in epileptic patients. Educating patients and parents about screening benefits and treatment outcomes can enhance adherence to treatment plans and improve overall patient outcomes. To our knowledge, this is the first study in the literature to describe the effect of *H. pylori* eradication on the serum level of an orally administered VPA in children with idiopathic generalized epilepsy.

## Figures and Tables

**Table 1:** Demographic and baseline characteristics.

Characteristics	Description (*n* = 100)
Gender	
Male	37 (37%)
Female	63 (63%)
Age (Yr.) Mean ± SD	5 ± 1.8
Age at diagnosis of epilepsy (Mean ± SD)	3.5 ± 1.2
Type of epilepsy	
Generalized tonic clonic	71 (71%)
Absence	29 (29%)
No. of epileptic attacks per month (Mean ± SD)	2.6 ± 1.5
Dose of valproic acid (mg/kg/day)	29.6 ± 10
Baseline valproic acid serum level (mcg/mL)	78.4 ± 17.7
Post-eradication valproic acid serum level (mcg/mL)	90.4 ± 18.7

**Table 2:** Comparison between *H. pylori*-positive and -negative groups.

	*H. pylori*		*p* Value
	Positive (*n* = 50)	Negative (*n* = 50)	
Gender			0.147
Male	15 (30%)	22 (44%)	
Female	35 (70%)	28 (56%)	
Age (Yr.)			0.613
Mean ± SD	5.1 ± 1.9	4.8 ± 1.7	
Median (IQR)	5 (3.6–6.4)	5 (3–5.9)	
Age at diagnosis of epilepsy (Yr.)	3.4 ± 1.2	3.6 ± 1.2	0.634
Type of epilepsy			**0.015**
Generalized tonic clonic seizures	41(82%)	30 (60%)	
Absence	9 (18%)	20 (40%)	
No. of epileptic attacks per month			0.401
Mean ± SD	3.1 ± 1.6	2.1 ± 1.2	
Median (IQR)	3 (2–4)	2 (1–3)	
GIT manifestations			
Abdominal pain	25 (50%)	12 (24%)	**0.007**
Nausea	11 (22%)	2 (4%)	**0.007**
Vomiting	18 (36%)	1 (2%)	**0.000**
Hematemesis	4 (8%)	0 (0%)	0.117
Melena	3 (6%)	0 (0%)	0.242
Diarrhea	3 (6%)	2 (4%)	0.646
Constipation	7 (14%)	8 (16%)	0.779
Hair changes			
Dry	26 (52%)	12 (24%)	**0.004**
Loose/Fall out	29 (58%)	18 (36%)	**0.028**
CBC			
Hemoglobin (g/dL)	10.8 ± 1.3	12.8 ± 2.1	**0.000**
MCV (μm^3^)	72.1 ± 8	77.9 ± 6.9	**0.000**
MCH (pg/ cell)	24.7 ± 2.8	26.5 ± 2.7	**0.001**
WBC (K/µL)	7.2 ± 2	6.7 ± 1.8	0.136
PLT (K/µL)	320.2 ± 85.5	307.1 ± 83.3	0.238
Ferritin (ng/mL)	17 ± 5.5	71.3 ± 55.1	**0.000**
TIBC (mcg/dL)	374 ± 36.5	360.7 ± 42.6	**0.001**

Bolded value = significant *p* value < 0.05.

**Table 3:** Valproic acid serum level before and after eradication therapy.

	*H. pylori*	
	Positive (*n* = 50)	Negative (*n* = 50)
Baseline valproic acid serum level (mcg/mL)		
Mean ± SD	79.9 ± 13.9	77.9 ± 13.1
Median (IQR)	77.5 (70–90)	67.5 (59–76)
Post-eradication valproic acid serum (mcg/mL)		
Mean ± SD	99.4 ± 11	85.3 ± 10.9
Median (IQR)	98.5 (87–102)	87.5 (79–93)
*p* value	**0.000**	0.142

Bolded value = significant *p* value < 0.05.

**Table 4:** Change in weight, appetite and activity after eradication therapy.

	*H. pylori*		*p* Value
	Positive (after Eradication) (*n* = 50)	Negative (*n* = 50)	
Weight change			0.016
No change	18 (36)	27 (54)	
Increased	27 (54)	16 (32)	
Decreased	5 (10)	7 (14)	
Appetite Change			0.005
No change	13 (26)	20 (40)	
Increased	23 (46)	12 (24)	
Decreased	14 (28)	18 (36)	
Activity			0.025
No change	21 (42)	29 (58)	
Increased	25 (50)	12 (24)	
Decreased	4 (8)	9 (18)	

Bolded value = significant *p* value < 0.05.

## Data Availability

The original contributions presented in this study are included in the article; further inquiries can be directed to the corresponding author.

## Supplementary Materials

The following supporting information can be downloaded at: https://www.mdpi.com/article/10.3390/children11101259/s1. Table S1. Comparison of valporic acid serum level % change (before and after treatment) and epidemiological findings according to *H. pylori* Ag in stool. Table S2. Comparison of valporic acid serum level % change and hematological changes according to *H. pylori* Ag in stool. Table S3. Comparison of valporic acid serum level % change and both the gender and nutritional status of the *H. pylori*-positive group. Table S4: Comparison of valporic acid serum level % change and both the gender and nutritional status of the *H. pylori*-negative group. Table S5. There was a statistically significance association between *H. pylori* eradication and the number of epileptic attacks per month (*p* value < 0.001).

## References

[1] Malfertheiner P., Camargo M.C., El-Omar E., Liou J.M., Peek R., Schulz C., Smith S.I., Suerbaum S. (2023). *Helicobacter pylori* infection. Nat. Rev. Dis. Primers.

[2] Lai H.H., Lai M.W. (2022). Treatment of Pediatric *Helicobacter pylori* Infection. Antibiotics.

[3] Gravina A.G., Priadko K., Ciamarra P., Granata L., Facchiano A., Miranda A., Dallio M., Federico A., Romano M. (2020). Extra-Gastric Manifestations of *Helicobacter pylori* Infection. J. Clin. Med..

[4] Mărginean C.D., Mărginean C.O., Meliț L.E. (2022). *Helicobacter pylori*-Related Extraintestinal Manifestations-Myth or Reality. Children.

[5] Ozturk A., Ozturk C.E., Ozdemirli B., Yucel M., Bahçebaşi T. (2007). *Helicobacter pylori* infection in epileptic patients. Seizure.

[6] Gorlé N., Bauwens E., Haesebrouck F., Smet A., Vandenbroucke R.E. (2021). *Helicobacter* and the Potential Role in Neurological Disorders: There Is More Than *Helicobacter pylori*. Front. Immunol..

[7] Kountouras J., Boziki M.K., Tzitiridou-Chatzopoulou M., Zavos C., Kazakos E. (2024). A potential effect of active *Helicobacter pylori* infection on the risk of multiple sclerosis. Neurol. Sci..

[8] Leta V., Klingelhoefer L., Longardner K., Campagnolo M., Levent H.Ç., Aureli F., Metta V., Bhidayasiri R., Chung-Faye G., Falup-Pecurariu C. (2023). Gastrointestinal barriers to levodopa transport and absorption in Parkinson’s disease. Eur. J. Neurol..

[9] Stillhart C., Vučićević K., Augustijns P., Basit A.W., Batchelor H., Flanagan T.R., Gesquiere I., Greupink R., Keszthelyi D., Koskinen M. (2020). Impact of gastrointestinal physiology on drug absorption in special populations—An UNGAP review. Eur. J. Pharm. Sci..

[10] Fiorini G., Bland J.M., Hughes E., Castelli V., Vaira D. (2015). A systematic review on drugs absorption modifications after eradication in *Helicobacter pylori* positive patients undergoing replacement therapy. J. Gastrointest. Liver Dis..

[11] Konstantinidou S.K., Argyrakopoulou G., Dalamaga M., Kokkinos A. (2023). The Effects of Bariatric Surgery on Pharmacokinetics of Drugs: A Review of Current Evidence. Curr. Nutr. Rep..

[12] Wohlt P.D., Zheng L., Gunderson S., Balzar S.A., Johnson B.D., Fish J.T. (2009). Recommendations for the use of medications with continuous enteral nutrition. Am. J. Health Syst. Pharm..

[13] Hant F.N., Bolster M.B. (2016). Drugs that may harm bone: Mitigating the risk. Cleve Clin. J. Med..

[14] Romoli M., Mazzocchetti P., D’Alonzo R., Siliquini S., Rinaldi V.E., Verrotti A., Calabresi P., Costa C. (2019). Valproic Acid and Epilepsy: From Molecular Mechanisms to Clinical Evidences. Curr. Neuropharmacol..

[15] Scheffer I.E., Berkovic S., Capovilla G., Connolly M.B., French J., Guilhoto L., Hirsch E., Jain S., Mathern G.W., Moshé S.L. (2017). ILAE classification of the epilepsies: Position paper of the ILAE Commission for Classification and Terminology. Epilepsia.

[16] Jones N.L., Koletzko S., Goodman K., Bontems P., Cadranel S., Casswall T., Czinn S., Gold B.D., Guarner J., Elitsur Y. (2017). Joint ESPGHAN/NASPGHAN Guidelines for the Management of *Helicobacter pylori* in Children and Adolescents (Update 2016). J. Pediatr. Gastroenterol. Nutr..

[17] Ertem D. (2013). Clinical practice: *Helicobacter pylori* infection in childhood. Eur. J. Pediatr..

[18] Behrangi E., Mansouri P., Agah S., Ebrahimi Daryani N., Mokhtare M., Azizi Z., Ramezani Ghamsari M., Rohani Nasab M., Azizian Z. (2017). Association between *Helicobacter Pylori* Infection and Alopecia Areata: A Study in Iranian Population. Middle East. J. Dig. Dis..

[19] Hudak L., Jaraisy A., Haj S., Muhsen K. (2017). An updated systematic review and meta-analysis on the association between *Helicobacter pylori* infection and iron deficiency anemia. Helicobacter.

[20] Lahner E., Virili C., Santaguida M.G., Annibale B., Centanni M. (2014). *Helicobacter pylori* infection and drugs malabsorption. World J. Gastroenterol..

[21] Aldhaleei W.A., Wallace M.B., Harris D.M., Bi Y. (2024). *Helicobacter pylori*: A concise review of the latest treatments against an old foe. Cleve Clin. J. Med..

[22] Centanni M., Gargano L., Canettieri G., Viceconti N., Franchi A., Delle Fave G., Annibale B. (2006). Thyroxine in goiter, *Helicobacter pylori* infection, and chronic gastritis. N. Engl. J. Med..

[23] Pierantozzi M., Pietroiusti A., Brusa L., Galati S., Stefani A., Lunardi G., Fedele E., Sancesario G., Bernardi G., Bergamaschi A. (2006). *Helicobacter pylori* eradication and l-dopa absorption in patients with PD and motor fluctuations. Neurology.

[24] Valiyaveettil A.N., Hamide A., Bobby Z., Krishnan R. (2005). Effect of anti-*Helicobacter pylori* therapy on outcome of iron-deficiency anemia: A randomized, controlled study. Indian J. Gastroenterol..

[25] De Caro C., Leo A., Nesci V., Ghelardini C., di Cesare Mannelli L., Striano P., Avagliano C., Calignano A., Mainardi P., Constanti A. (2019). Intestinal inflammation increases convulsant activity and reduces antiepileptic drug efficacy in a mouse model of epilepsy. Sci. Rep..

[26] Ghodke-Puranik Y., Thorn C.F., Lamba J.K., Leeder J.S., Song W., Birnbaum A.K., Altman R.B., Klein T.E. (2013). Valproic acid pathway: Pharmacokinetics and pharmacodynamics. Pharmacogenet. Genom..

[27] Löscher W. (2002). Basic pharmacology of valproate: A review after 35 years of clinical use for the treatment of epilepsy. CNS Drugs.

[28] Franceschi F., Annalisa T., Teresa D.R., Giovanna D., Ianiro G., Franco S., Viviana G., Valentina T., Riccardo L.L., Antonio G. (2014). Role of *Helicobacter pylori* infection on nutrition and metabolism. World J. Gastroenterol..

[29] Nweneka C.V., Prentice A.M. (2011). *Helicobacter pylori* infection and circulating ghrelin levels—A systematic review. BMC Gastroenterol..

