# Supplementary material for: Effect of Helicobacter pylori Eradication on Serum Level of Valproic Acid in Children with Idiopathic Generalized Epilepsy
Source: Children (Basel). 2024 Oct 18;11(10):1259. doi: 10.3390/children11101259 (PMC11506667; doi:10.3390/children11101259)
Supplement: Supplementary file 1 [file children-11-01259-s001.zip › children-3233543-supplementary.pdf]

Table S1. Comparison of valporic acid serum level % change (before and after treatment) and epidemiological finding according to *H. pylori* Ag in stool.

	**Valproic Acid Serum Level % Change**			
	***H. pylori* Ag in Stool** **+VE** **(*n* = 50)**		***H. pylori* Ag in Stool** **−VE** **(*n* = 50)**	
	**R**	***p***	**R**	***p***
**Age**	−0.160	0.266	0.103	0.476
**Age at diagnosis of epilepsy**	−0.017	0.909	0.168	0.245
**No. Of epileptic attack per month**	−0.301	**0.033**	−0.324	0.082

Table S1 shows that there was a statistically significance association with negative correlation between valproic acid serum level % change (before and after treatment) and number of epileptic attack per month (*p* value = 0.033, R value = −0.301) in *H. pylori* +ve Ag in stool. On the other hand, there was no statistically significant association between valproic acid serum level % change (before and after treatment) and age at diagnosis of epilepsy in both group, and there was no statistically significant association between valproic acid serum level % (before and after treatment) and number of epileptic attack per month in *H. pylori* −ve Ag in stool.

Table S2. Comparison of valporic acid serum level % change and hematological changes according to *H. pylori* Ag in stool.

	**Valproic Acid Serum Level % Change**			
	***H. pylori* Ag in Stool** **+VE** **(*n* = 50)**		***H. pylori* Ag in Stool** **−VE** **(*n* = 50)**	
	**R**	***p***	**R**	***p***
**Hb**	−0.074	0.611	0.265	0.063
**HCT**	−0.112	0.438	0.235	0.101
**MCV**	0.008	0.955	−0.021	0.886
**MCH**	0.080	0.581	−0.012	0.935
**RDW**	−0.074	0.612	0.059	0.682
**TLC**	−0.078	0.592	0.243	0.089
**Plt**	−0.107	0.460	−0.113	0.433
**TIBC**	0.228	0.112	−0.054	0.711
**Ferritin**	−0.166	0.249	0.146	0.311
**Transferrin Saturation**	−0.237	0.098	0.211	0.142

Table S2 shows that there was no statistically significance association between valproic acid serum level % change (before and after treatment) and CBC findings or iron profile in both groups.

Table S3. Comparison of valporic acid serum level % change and both Gender, and Nutritional status of *H. pylori* positive group.

	***H. pylori* Ag +VE**			**p Value**
	**Valporic Acid Serum Level % Change**			
	**Range**	**Mean ± SD**	**Median (IQR)**	
**Gender**				0.642
Male (n = 15)	8.2–50.7	22.2 ± 12.5	19.6 (12.2–26.3)	
Female (n = 35)	3.3 – 55.8	20.5 ± 11.2	20.5 (11.5–26)	
**Weight change**				0.063
No change (n = 18)	1.7–45.5	17.2 ± 10.3	15.8(9.7–20.2)	
Increased (n = 27)	1.98–48.3	19.8±9.9	18.5(13.1–25.5)	
Decreased (n = 5)	0.5–12.6	4.8 ± 2.8	4.4(2.7–5.7)	
**Appetite Change**				0.812
No change (n = 13)	1.9–27.1	11 ± 5.7	10.8(6.6–13.3)	
Increased (n = 23)	4.1–51.2	20.1±11.5	18.9(11.9–25.8)	
Decreased (n = 14)	1.7–25.3	10.4 ± 5.3	10.1(6.2–12.6)	
**Activity**				0.438
No change (n = 21)	2.3–48.8	18.4 ± 10.4	17.2(10.5–21.8)	
Increased (n = 25)	4.5–48.3	20±10.8	18.8(12.7–25.6)	
Decreased (n = 4)	0.4–9.3	3.5 ± 1.9	3.2(2–4.2)	

Table S3 showed that there was no statistically significance association between valporic acid serum level % change and Gender or nutritional status within *H.pylori* +ve Ag in stool group.

Table S4. Comparison of valporic acid serum level % change and both gender and nutritional status of *H. pylori* negative group.

	H. pylori **Ag –VE**			***p*** **Value**
	**Valporic Acid Serum Level % Change**			
	**Range**	**Mean ± SD**	**Median (IQR)**	
**Gender**				0.738
Male (*n* = 22)	3.8–52.6	20.1 ±11.9	18.9(11.6–25.6)	
Female (*n* = 28)	3.5–54	21.7 ± 11.8	20.8(12.7–26.1)	
**Weight change**				0.195
No change (*n* = 27)	2.9–42.3	16.5±8.6	16.4(9.5–20.7)	
Increased (*n* = 16)	6.4–53.2	21.5±13.9	19.5(11.6–26.1)	
Decreased (*n* = 7)	1.8–10.9	4.2±2.2	4.2(2.4–5.3)	
**Appetite Change**				0.792
No change (*n* = 20)	1.4–29.1	10.1±5.7	10.2(5.5–12.9)	
Increased (*n* = 12)	5.2–48.3	21.9±12.54	20.4(11.1–29.3)	
Decreased (*n* = 18)	1.4–29.1	10.1±5.7	10.2(5.5–12.9)	
**Activity**				0.584
No change (*n* = 29)	2.9–44.2	15.6±8.5	15.4(9.2–19.6)	
Increased (*n* = 12)	5.2–48.3	21.9±12.5	20.4(11.1–29.3)	
Decreased (*n* = 9)	0.7–13.9	4.9±.2.7	4.8(2.9–6.1)	

Table S4 showed that there was no statistically significance association between valporic acid serum level % change and both gender and Nutritional status within *H. pylori* negative group.

Table S5. Comparison of No. of epileptic attack per month according to *H. pylori* eradication.

	***H. pylori* Ag in Stool**<br><br>**+VE**<br>**(*n* = 50)**
**No. of epileptic attack per month before eradication**	
Range	2–5
Mean ± SD	3.1 ± 1.6
Median (IQR)	3 (2–4)
**No. of epileptic attack per month after eradication**	
Range	1–3
Mean ± SD	1.6 ± 1
Median (IQR)	2 (1–2)
***p* value**	**0.001**

Table S5 showed that there was a statistically significance association between *H. pylori* eradication and number of epileptic attack per month (p value < 0.001).
